# Effect of early feeding practices and eating behaviors on body composition in primary school children

**DOI:** 10.1007/s12519-022-00559-9

**Published:** 2022-06-06

**Authors:** Omneya Magdy Omar, Mohamed Naguib Massoud, Afaf Gaber Ibrahim, Nada Atef Khalaf

**Affiliations:** 1 Department of Pediatrics, Faculty of Medicine, Alexandria University, Alexandria, 21321 Egypt; 2 Department of Community Medicine and Public Health, Faculty of Medicine, Alexandria University, Alexandria, Egypt

**Keywords:** Blood pressure, Child eating behaviors, Childhood obesity, Feeding practices, Waist circumference

## Abstract

**Background:**

Understanding children’s feeding practices and eating behaviors is important to determine etiology of childhood obesity. This study aimed to explore the relationship between early feeding practices, eating behavior and body composition among primary school children.

**Methods:**

The data were collected from 403 primary school children. They were administered structured questionnaire, including sociodemographic characteristics, early feeding practices and Child’s Eating Behavior Questionnaire. Anthropometric and blood pressure (BP) measurements were performed.

**Results:**

Children with obesity and overweight showed higher food approach subscales and lower food avoidance subscales compared to a healthy and underweight child. Children who were exclusively or predominantly breast fed during the first 6 months had the lowest scores for the food approach subscales, food responsiveness (FR) and emotional overeating (EOE) and had the highest scores for the food avoidance subscales, satiety responsiveness (SR) and emotional under eating (EUE). Children who were introduced solid food after 6 months showed lower scores for FR, enjoyment of food and EOE but scored highest for SR, slowness in eating (SE) and EUE. All anthropometric measurements were positively correlated with all food approach subscales and negatively with SE, SR and food fussiness. All food approach subscales were positively correlated with BP percentiles. All food avoidance subscales were negatively correlated with both BP percentiles, except for EUE, which was negatively correlated with diastolic BP percentile only. Age, SR, SE and FR were predictors for child body mass index.

**Conclusion:**

Early feeding practices and eating behavior are considered as prevention approaches for obesity.

**Supplementary Information:**

The online version contains supplementary material available at 10.1007/s12519-022-00559-9.

## Introduction

Childhood overweight and obesity continue to be a universal health epidemic [1], with the prevalence of obesity in children increasing dramatically worldwide in under a generation [2]. Obesity in children certainly has a complex etiology and is most probably caused by several factors, ranging from hereditary to personal differences in a child’s eating behavior [3]. Nonetheless, several perinatal factors, including breastfeeding, have been associated with decreased risk of obesity in children [4]. Multiple hormone molecules that have an effect on fat and lean body mass formation seem to play a role in the development of obesity [5]. They also seem to promote appetite signaling, enhancing satiety responsiveness and lowering the risk of overeating in children [6].

The early childhood period is distinctive and critical given that eating behaviors develop during this time. This period provides the perfect opportunity for implementing obesity preventive initiatives [7]. The effect of eating behaviors on childhood obesity has been well described in the literature, which has shown that body mass index (BMI) was directly correlated with food approach subscales, but was inversely correlated with food avoidance subscales [8, 9].

The relationship between a faster rate of food intake and higher BMI and obesity has been well established [8, 10]. In fact, eating the very same meal over 30 minutes instead of 5 minutes promoted elevated concentrations of anorexigenic gut peptides and favored earlier satiety. As such, studies have recommended “eating slowly” for controlling food intake and thus body weight [11]. Emotional over/undereating may stem from the child’s incapacity to deal with surrounding stressors [10]. Indeed, approximately 30% of school-aged children who suffer from obesity engage in emotional eating, which has been positively associated with BMI [8]. With regard to food preference and rejection, evidence has shown that food pickiness and food neophobia are principal barriers to healthy eating traits [12]. Neophobic and picky children often present with unsatisfactory dietary diversities, with an occasional decrease in the type and number of foods accepted [13].

Increasing evidence has suggested that the intake of sugar-sweetened beverages is associated with increased body weight and elevated risk of medical problems [14]. Psychometric measures, such as the Child’s Eating Behavior Questionnaire (CEBQ), can be utilized to investigate eating behaviors that may play a role in the “obesity epidemic” currently experienced by the human population [9]. The current study primarily aimed to explore the relationship between eating behaviors with early feeding practices and body composition in school-aged children.

## Methods

**Fig. 1:**
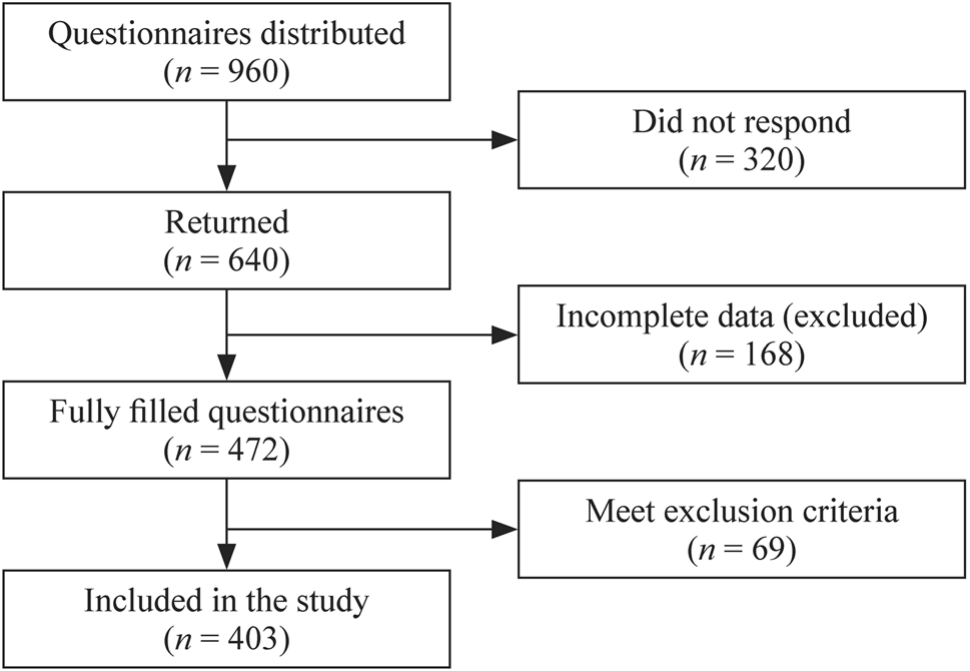
Flow chart of the studied population

A total of 960 questionnaires were distributed to primary school children of both sexes selected randomly from all eight educational districts throughout Alexandria governorate, among which 640 were returned, resulting in a response rate of 47.9%. Overall, 237 (16.97%) children were excluded, among whom 168 and 69 had incomplete data and satisfied the exclusion criteria, respectively. As such, 403 children [177 (43.9%) boys and 226 (56.1%) girls] were ultimately included in our final analysis (Fig. 1).

The following equation [15] was used to calculate the sample size.

$$N = \frac{{Z^{2}_{1 - a/2} \times P \times (1 - P)}}{{d^{2} }},$$

where *Z*^2^_1−*a*/2_ = 1.96 × 1.96, *P* = expected prevalence of overweight or obesity = 45% [16], *d* = degree of precision and *n* = 1.96 × 1.96 × 0.45 × 0.55**/**0.05 × 0.05 = 380 ≈ 400.

Multistage stratified random sampling was be used to obtain the calculated number of students. Overweight was defined as a BMI above the 1 standard deviation above the World Health Organization (WHO) growth standard median for age and sex, while obesity was defined as a BMI above 2 standard deviations above the WHO growth standard median for age and sex [17].

Exclusion criteria were children with chronic medical conditions, obesity due to endocrinal causes (e.g., Cushing syndrome, hypothyroidism and syndromic obesity) and chronic drug use leading to obesity (e.g., glucocorticoids, tricyclic antidepressants and antiepileptic drugs). Ethical approval for the study was obtained from the local Faculty of Medicine Ethics Committee and informed consent was obtained from the parents or guardians of each case.

Data regarding the child’s age in years, his/her gestational age, parental education and occupation, breast feeding duration (in months) and timing of solid food introduction (start of weaning) were collected. Children then were classified into four groups according to breastfeeding duration: those who never breastfed (BF), BF for 6 months or less, BF for 7–12 months and BF for more than 12 months. Feeding pattern during the first 6 months of life was categorized into exclusive breastfeeding, predominant breastfeeding, mixed feeding, complementary feeding and formula feeding. Solid food introduced was categorized according to time of introduction (i.e., before 4 months, between 4 and 6 months and after 6 months).

CEBQ is a 35-item questionnaire that assesses the following eight subscales of eating behavior: food responsiveness (FR, 5 items), enjoyment of food (EF, 4 items), desire to drink (DD, 3 items), emotional over eating (EOE, 4 items), slowness in eating (SE, 4 items), satiety responsiveness (SR, 5 items), food fussiness (FF, 6 items) and emotional under eating (EUE, 4 items) [18]. The initial four subscales (EF, FR, EOE and DD) can be characterized as “food approach” subscales showing positive tendencies for eating, whereas the other four subscales (SR, SE, FF and EUE) can be characterized as “food avoidance” subscales showing negative tendencies in food intake. EF and FR reflect various aspects of excessive responsiveness to external food cues, while EOE and EUE explore increased or decreased eating patterns in response to negative feelings, such as annoyance, loneliness, or worrying. DD is related to children’s tendency to drink frequently, sometimes coupled with elevated intakes of sugar-sweetened drinks, while SR reflects the child’s capability to reduce his food consumption after eating to control energy intake [19]. High SE scores suggest decreased eating speed resulting from the lack of enjoyment and interest in food, while FF reflects the rejection of a considerable amount of familiar and unfamiliar foods, decreasing the diversity of consumed foods. Each parent was asked to evaluate his/her child's eating behavior on a five-point Likert scale (never, rarely, sometimes, often and always; 1–5). Reports have shown that the CEBQ possesses adequate internal consistency, test − retest reliability and stability over time [20, 21]. The entire questionnaire was translated into Arabic and was approved by authorized translators. A pilot study was conducted (*n* = 40), after which the content validity index (assessed by six professors) was calculated (S-CVI/Ave = 0.913).

Anthropometric measures, including height (cm) and weight (kg), were recorded. *Z* scores were calculated using the WHO standards [17]. The same researcher took all anthropometric measurements with the same instruments. Height was measured using a stadiometer; weight was measured by pediatric scales. Waist circumference (WC) was measured at the uppermost lateral border of the hip crest with the child standing and breathing normally, while hip circumference (HC) was measured at the level of widest portion of buttocks [22]. WC and HC percentiles were calculated using charts based on age and sex. All circumferences were measured using a nonstretchable plastic tape with the children standing upright, their faces directed forward and both shoulders relaxed. The waist to hip ratio (WHR) was calculated for all participants. BMI was calculated as body weight in kilograms/height in meters^2^. Blood pressure (BP) was measured with children placed in a sitting position. Systolic and diastolic BP percentiles were calculated using a BP chart based on age and height. BP was categorized according to the American Academy of pediatrics updated definitions of BP categories and stages [23].

The data were analyzed using IBM SPSS software package version 20.0 (IBM Corp, Armonk, NY). Categorical data were represented as numbers and percentages. Continuous data were tested for normality by the Skewness test. Distributed data were expressed as range (minimum and maximum), mean and standard. Comparisons between groups were conducted using the Chi-square test (Monte Carlo correction) for categorical variables. An one-way ANOVA test was used for comparing the different studied groups and followed by Tukey’s post hoc test for pairwise comparison. The Pearson’s coefficient sed to determine correlations between two normally distributed quantitative variables. Linear regression analysis was used to identify the most independent factor affecting child BMI *Z* score. The level of significance for all statistical analyses was set at 5%.

## Results

The demographics of the children are presented in Table 1. Among the studied children, 22.6% and 24.1% were obese and overweight, while 50.1% and 3% were of healthy weight and underweight, respectively. Children with obesity and overweight showed higher food approach subscale scores and lower food avoidance subscale scores as compared to the other groups. In contrast, underweight children showed higher avoidance subscale scores and lower food approach subscale scores compared to the other groups. Children with obesity and overweight had significantly higher mean FR, EOE and EF scores compared to all other weight groups. Moreover, children with obesity and overweight had significantly higher mean DD scores compared to healthy weight children; however, although children with obesity and overweight had mean DD scores compared to underweight children, the difference was not significant. A significant difference in the mean SR scores was observed between all four weight groups (Table 2).

**Table 1:** Demographics of the analyzed samples (*N* = 403)

Variables	Total
Age (y)	
Min–max	5–14
Mean ± SD	9.1 ± 1.7
Sex, *n* (%)	
Male	177 (43.9)
Female	226 (56.1)
Education status of mother, *n* (%)	
Illiterate	40 (9.9)
Pre university	183 (45.4)
University	180 (44.7)
Education status of father, *n* (%)	
Illiterate	44 (10.9)
Pre university	191 (47.4)
University	168 (41.7)
Job of mother, *n* (%)	
Housewife	263 (65.3)
Working mother	140 (34.7)
Job of father, *n* (%)	
Manual worker	110 (27.3)
Non manual worker	293 (72.7)
Status at birth, *n* (%)	
Preterm	73 (18.1)
Full term	330 (81.9)
Breastfeeding duration, *n* (%)	
Never	127 (31.51)
Less than or 6 mon	94 (23.33)
From 7 to 12 mon	58 (14.39)
More than 12 mon	124 (30.7)
Pattern of feeding in the first 6 mon, *n* (%)	
Exclusive breastfeeding	61 (15.1)
Predominant breastfeeding	60 (14.9)
Mixed	133 (33)
Complementary feeding	22 (5.4)
Formula feeding	127 (31.5)
Time of solid food introduction	
Less than or 4 mon	82 (20.3)
From 5 to 6 mon	160 (39.7)
More than 6 mon	161 (40.0)
BMI *Z* score, mean ± SD	0.8 ± 1.7
Height *Z* score, mean ± SD	0.4 ± 1.0
Waist circumference (cm), mean ± SD	66.4 ± 8.0
Waist circumference percentile, mean ± SD	57.2 ± 22.7
Hip circumference (cm), mean ± SD	74.6 ± 9.5
Hip circumference percentile, mean ± SD	73.3 ± 25.9
WHR, mean ± SD	0.89 ± 0.55
SBP percentile, mean ± SD	62.7 ± 18.8
DBP percentile, mean ± SD	61.4 ± 17.8

*Min − max* minimum − maximum, *SD* standard deviation, *BMI* body mass index, *WHR* waist to hip ratio, *SBP* systolic blood pressure, *DBP* diastolic blood pressure

**Table 2:** Child’s Eating Behavior Questionnaire subscales according to categories of body mass index for age

Variables	Underweight (*n* = 13)	Healthy weight (*n* = 202)	Overweight (*n* = 97)	Obese (*n* = 91)	*F*	*P*
FR						
Mean ± SD	1.98 ± 1.08^a,b^	2.28 ± 0.73^a,b^	3.18 ± 0.92^c^	3.73 ± 0.84^d^	77.668	< 0.001^*^
95% CI of the mean	1.3310–2.6382	2.1779–2.3805	2.9901–3.3604	3.5509–3.8996		
EF						
Mean ± SD	2.65 ± 0.69^a,b^	3.02 ± 0.78^a,b^	3.87 ± 0.81^c,d^	4.12 ± 0.62^c,d^	61.138	< 0.001^*^
95% CI of the mean	2.2376–3.0700	2.9084–3.1262	3.7099–4.0375	3.9926–4.2492		
EOE						
Mean ± SD	1.71 ± 1.17^a,b^	2.03 ± 0.81^a,b^	2.97 ± 1.08^c^	3.35 ± 1.06^d^	51.387	0.001^*^
95% CI of the mean	1.0060–2.4170	1.9203–2.1440	2.7515–3.1867	3.1334–3.5754		
DD						
Mean ± SD	2.97 ± 1.04^a,b,c,d^	3.17 ± 1.05^a,b^	3.56 ± 0.98^a,c,d^	3.59 ± 1.09^a,c,d^	5.627	0.001^*^
95% CI of the mean	2.3456–3.6031	3.0193–3.3108	3.3655–3.7617	3.3623–3.8172		
SR						
Mean ± SD	4.14 ± 0.67^a^	3.47 ± 0.69^b^	2.45 ± 0.84^c^	2.09 ± 0.52^d^	112.252	< 0.001^*^
95% CI of the mean	3.7365–4.5405	3.3759–3.5686	2.2818–2.6213	1.9867–2.2023		
SE						
Mean ± SD	4.23 ± 0.56^a^	3.36 ± 0.91^b^	2.39 ± 0.84^c,d^	2.24 ± 0.65^c,d^	63.140	< 0.001^*^
95% CI of the mean	3.8904–4.5712	3.2369–3.4884	2.2246–2.5641	2.1068–2.3767		
EUE						
Mean ± SD	4.00 ± 0.74^a^	3.25 ± 0.87^b^	2.88 ± 0.78^c,d^	2.73 ± 0.64^c,d^	16.729	< 0.001^*^
95% CI of the mean	3.5553–4.4447	3.1315–3.3734	2.7220–3.0357	2.5974–2.8641		
FF						
Mean ± SD	2.99 ± 0.94^a,b,c^	2.81 ± 0.73^a,b,c^	2.61 ± 0.78^a,b,c,d^	2.40 ± 0.60^c,d^	8.020	< 0.001^*^
95% CI of the mean	2.4182–3.5561	2.7136–2.9151	2.4521–2.7678	2.2750–2.5235		

Post hoc multiple comparison using Tukey HSD method. Different superscript letters indicate statistically significant difference using pair wise sample. *FR* food responsiveness, *EF* enjoyment of food, *EOE* emotional over eating, *DD* desire to drink, *SR* satiety responsiveness, *SE* slowness in eating, *EUE* emotional under eating, *FF* food fussiness, *SD* standard deviation, *CI* confidence interval. ^*^Statistically significant (*P* ≤ 0.05)

Tables 3 and 4 summarize the CEBQ subscale scores according to the different parameters. No significant difference in the mean of any eating behavior subscales was observed according to sex, except for the FR subscale wherein females had higher mean scores than males. The age groups were classified according to the age distribution curve of the studied sample. Full-term children showed significantly higher mean EF and DD subscale scores as compared to preterm children, whereas preterm children showed significantly higher mean SR and SE subscale scores compared to full-term children. The mean SR scores were the lowest among children with illiterate fathers and mothers and were the highest among those with university-educated mothers. The mean FF scores were the lowest among those with illiterate fathers and were the highest among those with university-educated fathers. Children who were never BF scored higher on the food approach subscales FR and EOE, but scored lowest on the food avoidance subscale SR. On the contrary, those who were BF > 12 months scored the highest on SR and lowest on FR and EOE. Moreover, children who were exclusively or predominantly BF during the first 6 months of life had highest scores for the food avoidance subscales SR and EUE and lowest scores for the food approach subscales FR and EOE. On the other hand, those who were formula fed during the first 6 months of life showed higher scores for the food approach subscales FR and EOE. Children who were introduced solid food after 6 months showed lower scores for the food approach subscales FR, EF and EOE, but scored the highest on the food avoidance subscale SR, SE and EUE.

**Table 3:** Child’s Eating Behavior Questionnaire subscales according to gender, age of children and paternal education

	Gender			*P*	Age (y)			*P*	Status at birth		*P*	Education status of father				Education status of mother			
Variables		Male (*n* = 177)	Female (*n* = 226)		5–8 (*n* = 143)	> 8-< 11 (*n* = 185)	11–14 (*n* = 75)		Preterm (*n* = 73)	Full term (*n* = 330)		Illiterate (*n* = 44)	Pre university (*n* = 191)	University (*n* = 168)	*P*	Illiterate (*n* = 40)	Pre university (*n* = 183)	University (*n* = 180)	*P*
FR		2.80 ± 1.08	2.82 ± 0.98	0.043^*^	2.77 ± 1.06	2.84 ± 1.04	2.82 ± 0.91	0.794	2.67 ± 1.17	2.84 ± 0.98	0.186	2.99^a^ ± 1.04	2.77^a^ ± 0.98	2.82^a^ ± 1.06	0.432	2.65^a^ ± 0.99	2.86^a^ ± 1.0	2.80^a^ ± 1.06	0.499
EF		3.46 ± 0.88	3.46 ± 0.93	0.531	3.38 ± 0.97	3.48 ± 0.87	3.57 ± 0.88	0.314	3.27 ± 0.94	3.50 ± 0.90	0.048^*^	3.64^a^ ± 0.91	3.40^a^ ± 0.96	3.48^a^ ± 0.84	0.274	3.59^a^ ± 0.88	3.45^a^ ± 0.95	3.44^a^ ± 0.87	0.613
EOE		2.61 ± 1.15	3.46 ± 0.93	0.087	2.49 ± 1.12	2.59 ± 1.11	2.54 ± 1.15	0.762	2.41 ± 1.18	2.58 ± 1.10	0.254	2.77^a^ ± 1.23	2.51^a^ ± 1.09	2.53^a^ ± 1.11	0.374	2.62^a^ ± 1.0	2.54^a^ ± 1.14	2.53^a^ ± 1.12	0.907
DD		3.33 ± 1.01	3.36 ± 1.10	0.127	3.33 ± 1.00	3.40 ± 1.06	3.28 ± 1.18	0.693	3.11 ± 1.03	3.41 ± 1.06	0.029^*^	3.34^a^ ± 1.12	3.35^a^ ± 0.99	3.35^a^ ± 1.13	0.998	3.34^a^ ± 0.96	3.40^a^ ± 0.97	3.30^a^ ± 1.17	0.635
SR		2.82 ± 0.99	3.03 ± 0.90	0.051	2.92 ± 0.95	2.99 ± 1.00	2.83 ± 0.76	0.408	3.13 ± 1.06	2.89 ± 0.91	0.048^*^	2.55^b^ ± 0.76	2.98^a^ ± 0.97	2.99^a^ ± 0.94	0.014^*^	2.62^b^ ± 0.92	2.91^a,b^ ± 0.92	3.03^a^ ± 0.97	0.037^*^
SE		2.71 ± 0.97	3.06 ± 1.01	0.489	3.02 ± 1.08	2.84 ± 0.95	2.84 ± 0.99	0.235	3.17 ± 1.00	2.85 ± 1.00	0.013^*^	2.62^a^ ± 1.00	2.93^a^ ± 1.08	2.95^a^ ± 0.91	0.135	2.77^a^ ± 0.99	2.87^a^ ± 1.06	2.97^a^ ± 0.95	0.440
EUE		2.96 ± 0.85	3.15 ± 0.83	0.940	3.09 ± 0.88	3.09 ± 0.87	2.96 ± 0.70	0.435	3.04 ± 0.93	3.08 ± 0.83	0.757	2.92^a^ ± 0.85	3.01^a^ ± 0.85	3.18^a^ ± 0.83	0.082	2.83^a^ ± 0.75	3.04^a^ ± 0.86	3.15^a^ ± 0.85	0.087
FF		2.63 ± 0.70	2.71 ± 0.77	0.295	2.76 ± 0.71	2.64 ± 0.77	2.62 ± 0.71	0.247	2.71 ± 0.70	2.67 ± 0.75	0.653	2.42^b^ ± 0.63	2.68^a,b^ ± 0.76	2.75^a^ ± 0.74	0.032^*^	2.75^a^ ± 0.75	2.60^a^ ± 0.72	2.74^a^ ± 0.75	0.185

*F* for ANOVA test, pairwise comparison bet; each 2 groups were done using post hoc test (Tukey). Means with common letters are not significant (i.e., means with different superscript letters are significant). *FR* food responsiveness, *EF* enjoyment of food, *EOE* emotional over eating, *DD* desire to drink, *SR* satiety responsiveness, *SE* slowness in eating, *EUE* emotional under eating, *FF* food fussiness. ^*^Statistically significant (*P* ≤ 0.05)

**Table 4:** Child’s Eating Behavior Questionnaire subscales according to breast feeding duration, pattern of feeding in the first 6 months and time of solid food introduction

Variables	Breast feeding duration				*P*	Pattern of feeding in the first 6 mon				*P*	Time of solid food introduction			*P*
	Never (*n* = 127)	Less than or 6 mon (*n* = 94)	From 7 to 12 mon (*n* = 58)	More than 12 mon (*n* = 124)		Predominant breastfeeding (*n* = 60)	Mixed (*n* = 133)	Complementary feeding (*n* = 22)	Formula feeding (*n* = 127)		Less than or 4 mon (*n* = 82)	From 5 to 6 mon (*n* = 160)	More than 6 mon (*n* = 161)	
FR	3.04^a^ ± 1.09	2.76^a,b^ ± 1.00	2.82^a,b^ ± 0.96	2.61^b^ ± 0.96	0.011^*^	2.52^b^ ± 0.93	2.82^a,b^ ± 0.98	2.85^a,b^ ± 1.01	3.04^a^ ± 1.09	0.008^*^	3.05^a^ ± 1.07	2.99^a^ ± 0.99	2.51^b^ ± 0.96	< 0.001^*^
EF	3.61^a^ ± 0.95	3.46^a^ ± 0.92	3.38^a^ ± 0.83	3.34^a^ ± 0.87	0.102	3.35^a^ ± 0.86	3.44^a^ ± 0.90	3.36^a^ ± 1.05	3.61^a^ ± 0.95	0.194	3.66^a^ ± 0.90	3.66^a^ ± 0.92	3.16^b^ ± 0.83	< 0.001^*^
EOE	2.79^a^ ± 1.19	2.48^a,b^ ± 1.07	2.47^a,b^ ± 1.11	2.38^b^ ± 1.04	0.021^*^	2.20^b^ ± 0.97	2.63^a,b^ ± 1.03	2.55^a,b^ ± 1.09	2.79^a^ ± 1.19	0.001^*^	2.92^a^ ± 1.18	2.71^a^ ± 1.06	2.19^b^ ± 1.04	< 0.001^*^
DD	3.35^a^ ± 1.11	3.37^a^ ± 1.07	3.33^a^ ± 0.94	3.35^a^ ± 1.07	0.996	3.34^a^ ± 1.29	3.37^a^ ± 0.96	3.38^a^ ± 0.83	3.35^a^ ± 1.11	0.999	3.36^a^ ± 1.15	3.46^a^ ± 0.96	3.24^a^ ± 1.10	0.166
SR	2.74^b^ ± 0.97	2.91^a,b^ ± 0.85	3.01^a,b^ ± 0.90	3.13^a^ ± 0.98	0.013^*^	3.23^a^ ± 0.74	2.88^a,b^ ± 0.98	3.10^a,b^ ± 0.98	2.74^b^ ± 0.97	0.005^*^	2.75^b^ ± 0.98	2.64^b^ ± 0.83	3.33^a^ ± 0.90	< 0.001^*^
SE	2.71^a^ ± 1.03	2.98^a^ ± 0.99	2.94^a^ ± 0.89	3.02^a^ ± 1.02	0.073	3.13^a^ ± 1.05	2.92^a^ ± 0.95	2.99^a^ ± 0.99	2.71^a^ ± 1.03	0.078	2.63^b^ ± 1.00	2.70^b^ ± 0.89	3.25^a^ ± 1.02	< 0.001^*^
EUE	2.95^a^ ± 0.79	3.08^a^ ± 0.81	3.28^a^ ± 0.90	3.08^a^ ± 0.88	0.114	3.23^a,b^ ± 0.89	2.99^b^ ± 0.84	3.0^a,b^ ± 0.81	2.95^b^ ± 0.79	0.011^*^	2.83^b^ ± 0.73	3.01^b^ ± 0.82	3.25^a^ ± 0.89	0.001^*^
FF	2.65^a,b^ ± 0.62	2.90^a^ ± 0.75	2.69^a,b^ ± 0.74	2.53^b^ ± 0.81	0.003^*^	2.27^d^ ± 0.65	2.97^a^ ± 0.82	3.0^a,b^ ± 0.50	2.65^b,c^ ± 0.62	< 0.001^*^	2.75^a^ ± 0.76	2.62^a^ ± 0.76	2.70^a^ ± 0.71	0.419

*F* for ANOVA test, pairwise comparison bet; each 2 groups were done using post hoc test (Tukey). Means with common letters are not significant (i.e., means with different superscript letters are significant). *FR* food responsiveness, *EF* enjoyment of food, *EOE* emotional over eating, *DD* desire to drink, *SR* satiety responsiveness, *SE* slowness in eating, *EUE* emotional under eating, *FF* food fussiness. ^*^Statistically significant (*P* ≤ 0.05)

Correlation analysis among different subscales suggested that the three food approach subscales (EOE, FR and EF) and all food avoidance subscales (SR, SE, FF, EUE) tended to be negatively inter-correlated. Moreover, positive inter-correlations were observed among the “food approach” subscales (EOE, FR, EF and DD). Two of the food avoidance subscales (SE and SR) had a positive correlation with all food avoidance subscales, while FF and EUE had a positive correlation with SE and SR. No correlation was observed between DD and all food avoidance subscales and between EUE and FF (Table 5).

**Table 5:** Pearson’s correlations between Child’s Eating Behavior Questionnaire subscales

Variables	Items	EF	EOE	DD	SR	SE	EUE	FF
FR	*r*	0.739	0.713	0.272	– 0.655	– 0.473	– 0.269	– 0.297
	*P*	< 0.001^*^	< 0.001^*^	< 0.001^*^	< 0.001^*^	< 0.001^*^	< 0.001^*^	< 0.001^*^
EF	*r*		0.565	0.173	– 0.647	– 0.552	– 0.234	– 0.346
	*P*		< 0.001^*^	< 0.001^*^	< 0.001^*^	< 0.001^*^	< 0.001^*^	< 0.001^*^
EOE	*r*			0.172	– 0.577	– 0.486	– 0.370	– 0.187
	*P*			0.001^*^	< 0.001^*^	< 0.001^*^	< 0.001^*^	< 0.001^*^
DD	*r*				– 0.090	– 0.021	0.054	0.000
	*P*				0.072	0.677	0.282	0.995
SR	*r*					0.675	0.366	0.399
	*P*					< 0.001^*^	< 0.001^*^	< 0.001^*^
SE	*r*						0.336	0.382
	*P*						< 0.001^*^	< 0.001^*^
EUE	*r*							0.087
	*P*							0.083

*FR* food responsiveness, *EF* enjoyment of food, *EOE* emotional over eating, *DD* desire to drink, *SR* satiety responsiveness, *SE* slowness in eating, *EUE* emotional under eating, *FF* food fussiness, *r* Pearson coefficient. ^*^Statistically significant (*P* ≤ 0.05)

The food approach subscales (FR, EF, EOE and DD) tended to be positively correlated with all measured anthropometric measurements, except for WHR. The food avoidance subscales (SR, SE and FF) were negatively correlated with all measured anthropometric measurements, except WHR, while the EUE tended to be positively correlated with WHR and negatively correlated with all other measured anthropometric measurements. All food approach subscales were positively correlated with BP percentiles. Moreover, all food avoidance subscales were negatively correlated with both BP percentiles, except for EUE, which was negatively correlated with diastolic BP percentile, but not with systolic BP percentile. BMI *Z* scores tended to be positively correlated with all food approach subscales, BP percentiles and measured anthropometric measurements, except for WHR, but negatively correlated with all food avoidance subscales (Table 6).

**Table 6:** Correlations between Child’s Eating Behavior Questionnaire subscales and children physical examination

		Circumference percentile		BP percentile		Waist/hip ratio	Height SD	BMI *Z* score
Variables	Items	Waist	Hip	Systolic	Diastolic			
FR	*r*	0.512	0.445	0.314	0.247	0.077	0.237	0.416
	*P*	< 0.001^*^	< 0.001^*^	< 0.001^*^	< 0.001^*^	0.124	< 0.001^*^	< 0.001^*^
EF	*r*	0.428	0.464	0.314	0.278	– 0.036	0.212	0.375
	*P*	< 0.001^*^	< 0.001^*^	< 0.001^*^	< 0.001^*^	0.468	< 0.001^*^	< 0.001^*^
EOE	*r*	0.444	0.381	0.138	0.255	0.069	0.313	0.395
	*P*	< 0.001^*^	< 0.001^*^	0.005^*^	< 0.001^*^	0.167	< 0.001^*^	< 0.001^*^
DD	*r*	0.181	0.159	0.164	0.147	– 0.002	0.119	0.114
	*P*	< 0.001^*^	0.001^*^	0.001^*^	0.003^*^	0.975	0.017^*^	0.022^*^
SR	*r*	– 0.488	– 0.502	– 0.298	– 0.344	0.005	– 0.201	– 0.452
	*P*	< 0.001^*^	< 0.001^*^	< 0.001^*^	< 0.001^*^	0.924	0.001^*^	< 0.001^*^
SE	*r*	– 0.407	– 0.438	– 0.247	– 0.286	0.011	– 0.151	– 0.412
	*P*	< 0.001^*^	< 0.001^*^	< 0.001^*^	< 0.001^*^	0.821	0.002^*^	< 0.001^*^
EUE	*r*	– 0.284	– 0.327	– 0.045	– 0.148	0.105	– 0.124	– 0.241
	*P*	< 0.001^*^	< 0.001^*^	0.366	0.003^*^	0.035^*^	0.013^*^	< 0.001^*^
FF	*r*	– 0.206	– 0.254	– 0.295	– 0.242	0.086	– 0.155	– 0.164
	*P*	< 0.001^*^	< 0.001^*^	< 0.001^*^	< 0.001^*^	0.085	0.002^*^	0.001^*^
Waist circumference percentile	*r*		0.689	0.327	0.474	0.270	0.365	0.510
	*P*		< 0.001^*^	< 0.001^*^	< 0.001^*^	< 0.001^*^	< 0.001^*^	< 0.001^*^
Hip circumference percentile	*r*			0.233	0.382	– 0.208	0.403	0.491
	*P*			< 0.001^*^	< 0.001^*^	< 0.001^*^	< 0.001^*^	< 0.001^*^
Systolic BP percentile	*r*				0.251	0.112	0.114	0.164
	*P*				< 0.001^*^	0.025^*^	0.023^*^	0.001^*^
Diastolic BP percentile	*r*					0.049	0.297	0.331
	*P*					0.330	< 0.001^*^	< 0.001^*^
Waist/hip ratio	*r*						– 0.143	– 0.078
	*P*						0.004^*^	0.118
Height *Z* score	*r*							0.176
	*P*							< 0.001^*^

*FR* food responsiveness, *EF* enjoyment of food, *EOE* emotional over eating, *DD* desire to drink, *SR* satiety responsiveness, *SE* slowness in eating, *EUE* emotional under eating, *FF* food fussiness, *BP* blood pressure, *BMI* body mass index, *SD* standard deviation, *r* Pearson coefficient. ^*^Statistically significant (*P* ≤ 0.05)

Using linear regression models, our results showed that each CEBQ subscale significantly predicted child BMI *Z* score. For each unit increase in EF, EOE, DD and FR, child BMI *Z* score increased by 0.715 (*P* < 0.001), 0.615 (*P* < 0.001), 0.187 (*P* = 0.022) and 0.706 (*P* < 0.001), respectively. On the other hand, for each unit increase in SR, SE, FF and EUE, child BMI *Z* scores decreased by 0.830 (*P* < 0.001), 0.711 (*P* < 0.001), 0.385 (*P* = 0.001) and 0.494 (*P* < 0.001), respectively (Supplementary Table 1). Multiple regression analysis adjusting for child age, sex, birth status, feeding pattern during the first 6 months, time of solid food introduction and eating behavior subscales showed that SR (*P* = 0.012), SE (*P* = 0.002), FR (*P* = 0.031) and age (*P* = 0.004) were predictors of child BMI *Z* score, explaining 26% of the variance in the model (Supplementary Table 2).

## Discussion

Among the children included in the current study, 22.6%, 24.1%, 50.1% and 3% were obese, overweight, healthy weight and underweight, respectively. Interestingly, the prevalence of overweight and obesity in this study was higher than that reported in a 2014 Egyptian study by El-Shafie et al. [24] who reported 16.8% and 9% prevalence rates for overweight and obesity among Alexandria governorate children, respectively. The highest crude prevalence of childhood obesity in the WHO European region was observed in Mediterranean countries in 2016, ranging from 7.6% to 13.8%. Fighting childhood obesity is challenging and there are many preventive programs created by countries. For example, Malta controlled balance and micronutrient intake of at least one meal per day of all school kids. In addition, Italy started to use media, brochures and education in schools and in health care facilities [25].

The CEBQ is an essential tool for assessing eating behaviors in children. As such, the results obtained herein could be valuable in understanding the etiology of overweight and obesity in primary school children with regard to their eating behaviors. The current study found that food approach behaviors were positively associated with the risk of being overweight/obese but were negatively associated with the risk of being underweight. Increased food avoidance scores, on the other hand, were associated with a lower risk for overweight/obesity and greater risk for underweight.

Studies have reported that overweight children have a notable interest in food and a more prominent response capacity to the effects of external food cues, such as taste, color and smell [26, 27]. The significant differences in FR and EF subscale scores between BMI percentile weight categories observed in the present study were consistent with those reported in the previous studies [26, 28], indicating that children with higher BMI were more responsive to environmental food cues. Similarly, Power et al. [29] in 2020, in a sample of Hispanic children from low-income families, reported that FR was positively associated with child weight status, whereas SR was negatively associated with the same.

The present study found that higher BMI was positively associated with the EOE subscale and was inversely associated with the EUE subscale, which is consistent with the findings presented in previous studies [21, 26, 30]. Moreover, in a study of 520 healthy children between the ages of 2–12 years, Sanlier et al. [31] reported that obese children had significantly higher average EOE subscores relative to underweight, normal and overweight children. The current results are also consistent with those published by Saphić et al. [32] in 2019, which reported that BMI *Z* scores increased linearly with the EOE subscale and decreased with the EUE subscale in a sample of children aged 3–10 years. The research in children and adolescents has demonstrated a relationship between maladaptive emotional regulation strategies and emotional eating [33, 34]. Given the assumption that high energy density foods may have the ability to improve negative emotions or stress, emotional eating leads to an unhealthy diet. Therefore, emotional eating boosts the consumption of sweet or fatty foods, otherwise known as “comfort food” [35]. These findings contradict those presented by McCarthy et al. [36] who reported no significant associations between the EUE and EOE subscales and BMI. This difference could be explained by the fact that the children were too young (2 years of age) to demonstrate any aberrant eating patterns in reaction to emotional stimuli. Studies have suggested that younger children preserve the more natural response to emotional or stressful situations, which includes a decrease in appetite. As such, EOE can be considered an abnormal response in young children [37], while EUE is thought to be a more common response to stressful situations in young children [18]. The current study found that DD was positively correlated with BMI. Although Quah et al. [38] reported that higher DD subscale scores at year 6 were associated with overweight in children, Sanlier et al. [31] and Domoff et al. [21] reported no association between BMI *Z* score and the DD subscale. The mechanism by which DD may be associated with weight status requires further investigation. On the one hand, it could be a response to desire something in the mouth, wherein if such a person is offered caloric beverages, their energy intake will result in a positive energy balance [39]. On the other hand, it could be a specific desire for high caloric drinks. Another potential explanation could be that thirst is associated with weight gain as a consequence of snack intake considering that salt, which is often present in savory snacks, increases thirst, with studies showing an association between salt and overall fluid and sweet drinks intake in children aged 4–18 years old [40].

In the current study, both SR and SE were negatively correlated with BMI *Z* scores, confirming the notion that a lower satiety response makes children less capable of regulating food consumption, thereby promoting excess weight gain [18, 32]. This finding is consistent with that reported in a study carried out in Southern Brazil on 335 children aged 6–10 years [26]. In addition, a study conducted in 2018 among Latino children aged 5–11 years reported that child BMI was negatively associated with SR and SE [41]. Moreover, a study carried out in a sample of Hispanic children from low-income families reported a negative relationship between SR and weight status [29].

The current study found that FF was negatively correlated with children’s BMI *Z* scores, which is consistent with that presented in other studies [21, 36]. Moreover, the results reported by Boswell et al. [28] in a sample of 977 Australian children were consistent with those presented in the current study, wherein FF was negatively associated with BMI. In contrast, studies by Spahić et al. [32] in 2019 and by dos Passos et al. [26] in 2015 showed no association between BMI percentile weight categories and FF. The results by Finistrella et al. [12] which showed that picky eaters had a higher probability of obesity, were also inconsistent with the present study’s findings. They suggested that one possible causal mechanism could be that pickiness and neo-phobia might decrease the child’s dietary variation, especially fruit and vegetable intake, which might be substituted by processed foods, leading to the development of obesity. The inconsistent results could have been attributed to differences in sample recruitment and methods used to evaluate pickiness. Given the lack of definite information regarding pickiness and its relation to weight status, further investigations using validated measures in larger cohorts and those at risk of obesity are required.

The current study observed no significant difference in mean CEBQ subscale scores according to different age groups and sex, except for the FR subscale, wherein females had higher mean scores than males. This is comparable to the results presented in a study conducted in Saudi Arabia [42] on children aged 2–6 years, which revealed that age and sex had no significant effect on eating behaviors, except for FR, where males had higher FR scores than females. Moreover, Sanlier et al. [31] and dos Passos [26] reported that males had greater DD scores than females, with no difference in other eating behavior scores according to sex. Sanlier et al. [31] reported that the preschool group had higher SR, SE and EUE scores than the school group, with no difference in other eating behavior scores according to age. Moreover, dos Passos [26] reported that eating behavior was very similar across all age groups and that only the SE score showed a significant decrease with increasing age. This discrepancy in the results may be attributed to differences in age groups and social and cultural backgrounds among the studied samples.

To the best of our knowledge, this study has been the first to examine the association between birth status (preterm/full-term) and CEBQ subscales. However, despite using a different tool (the Children’s Eating Difficulties Questionnaire), Migraine et al. [43] had previously reported that preterm children had a worse drive-to-eat score compared to full-term children, which is consistent with the current results. Also, a 2016 study by Johnson et al. [44] on 1130 preterm and 1255 full-term children used a validated eating behavior questionnaire to assess the presence of eating difficulties across four domains (i.e., refusal/picky eating, oral motor problems, oral hypersensitivity and eating behavior problems) reported that preterm born children were at increased risk for refusal of eating and picky eating problems. In the present study, higher maternal and paternal education was associated with higher food avoidance subscale SR scores, a finding consistent with that presented in a study conducted in 2016, which reported that higher maternal education was associated with a higher SR at age 7 years [45]. The present study reported an insignificant association between the FR and EOE subscales and parental education. Contrarily, a study conducted in 2021 on 169 participants aged between 6 and 10 years in Alabama reported that children born to mothers with higher educational level showed significantly lower mean FR and EOE subscale scores as compared to those born to mothers with lower education level [46]. This is highlight that greater educational achievement among mothers may decrease their children’s vulnerability to obesogenic environment.

In the present study, children who were never BF scored higher on the food approach subscales FR and EOE, but scored the lowest on the food avoidance subscale SR. However, those who were BF > 12 months scored the highest on SR and the lowest on FR and EOE. Moreover, children who were exclusively or predominantly BF during the first 6 months of life had highest scores for the food avoidance subscales SR and EUE and lowest scores for the food approach subscales FR and EOE. Indeed, a study conducted in 2012 reported that children who were exclusively breastfed for 3–6 months scored higher on FR than those who received exclusive breastfeeding for at least 6 months [47]. Another study in 2018 reported that breastfeeding for less than 6 months was associated with decreased EF and increased FF [28]. Children who were introduced solid food after 6 months showed lower scores for the food approach subscales FR, EF and EOE, but scored the highest on the food avoidance subscale SR, SE and EUE. In agreement with the current results, Škledar et al. [48] found that children who start complementary feeding before sixth months of age were 2.46 times more likely to become overweight/obese.

To the best of our knowledge, this has been the first study to examine the correlation between CEBQ subscales and different child physical examination parameters, including blood pressure (WC percentile, HC percentile, systolic BP and diastolic BP percentiles, WHR and height *Z* scores). Accordingly, a positive correlation was found between all food approach subscales and all physical examination entities, except for WHR, whereas the food avoidance subscales had a negative correlation with most physical examination entities. This confirms that eating behavior can affect obesity and its consequences. Also, Dalrymple et al. [49] reported that FR and EF were associated with higher WC, weight-for-age, weight-for-height and BMI *Z* scores and higher odds of obesity in three-year-old children of mothers with obesity. In contrast, SE and SR were inversely associated with the same measures of body composition, suggesting that these traits are protective against an obesogenic environment. After adjusting for child age, sex and status at birth, feeding pattern during the first 6 months, time of solid food introduction and eating behavior subscales in regression analysis, only age, SR, SE and FR were identified as eating behavior traits significantly predictive of child BMI *Z* scores. This result is comparable to that presented by Boswell et al. [28] who showed that SR, FR and child age were significant predictors of BMI. The current findings suggest that strategies preventing obesity should focus on FR as an obesity promoting trait and SR and SE as obesity reducing traits. Identifying and classifying children with obesity as early as possible is important, as is identifying comorbid conditions [50]. The recent coronavirus disease 2019 pandemic has accelerated the treatment process through information technology/technological supports, which are useful in the management of chronic patients [51].

The current study has some limitations worth noting. Some factors that might play a role in childhood obesity, such as maternal obesity, pregnancy weight gain of the mother and weight gain of infants during the first months of life, were not included in the analysis. Moreover, given that information on infant feeding was obtained long after the time of actual breastfeeding, recall bias could have been a concern, granted that not all information was available from children’s medical charts. The use of questionnaire data to evaluate eating behaviors can also be a limitation given that it reflects parental perception of children’s behavior rather than objectively measured behavior. However, the strength of this research lies in its adjustment for a large number of confounding factors that may influence eating behavior and childhood obesity. The large number of anthropometric measurements and physical findings emphasizes the effects of obesity on the development cardiovascular risk factors and metabolic syndrome. The strengths of the associations found in the current study population suggest the need for future trials on larger samples.

In summary, we found that food approach eating behaviors were associated with obesity and measures of body composition. Early feeding practices, prematurity and parenteral education could affect eating behaviors.

## Supplementary Information

Below is the link to the electronic supplementary material.

Supplementary file 1 (DOCX 18 KB)

## Data Availability

All data used are included in this article. Further data that support the findings of this study are available from the corresponding author upon reasonable request by mail.
