# Supplementary material for: Effect of early feeding practices and eating behaviors on body composition in primary school children
Source: World J Pediatr. 2022 Jun 6;18(9):613–23. doi: 10.1007/s12519-022-00559-9 (PMC9169027; doi:10.1007/s12519-022-00559-9)
Supplement: Supplementary file 1 — (DOCX 18 KB) [file 12519_2022_559_MOESM1_ESM.docx]

**Supplementary Table 1.** Linear regression analysis for BMI *Z* scores and CEBQ subscales

| **Coefficients^a^** | | | | | | | |
| --- | --- | --- | --- | --- | --- | --- | --- |
| **Model** | **Unstandardized coefficients** | | **Standardized coefficients** | ***t*** | **Sig.** | **95% confidence interval for *B*** | |
|  | ***B*** | **SE** | **Beta** |  |  | **LL** | **UL** |
| **FR** | 0.706 | 0.077 | 0.416 | 9.167 | ˂ 0.001 | 0.555 | 0.857 |
| **EF** | 0.715 | 0.088 | 0.375 | 8.092 | ˂ 0.001 | 0.542 | 0.889 |
| **EOE** | 0.615 | 0.071 | 0.395 | 8.617 | ˂ 0.001 | 0.475 | 0.755 |
| **DD** | 0.187 | 0.081 | 0.114 | 2.307 | 0.022 | 0.028 | 0.346 |
| **SR** | -0.830 | 0.082 | -0.452 | -10.141 | ˂ 0.001 | -0.990 | -0.669 |
| **SE** | -0.711 | 0.079 | -0.412 | -9.054 | ˂ 0.001 | -0.866 | -0.557 |
| **EUE** | -0.494 | 0.100 | -0.241 | -4.964 | ˂ 0.001 | -0.690 | -0.299 |
| **FF** | -0.385 | 0.115 | -0.164 | -3.337 | 0.001 | -0.612 | -0.158 |

*FR* food responsiveness, *EF* enjoyment of food, *EOE* emotional over-eating, *DD* desire to drink, *SR* satiety responsiveness, *SE* slowness in eating, *EUE* emotional under-eating, *FF* food fussiness, *LL* lower limit, *UL* upper limit, *BMI* body mass index, *SD* standard deviation, *CEBQ* Child’s Eating Behavior Questionnaire. ^a^Dependent variable: BMI SD

**Supplementary Table 2.** Variables predictive of child BMI *Z* score in multiple regression

| **Coefficients^a^** | | | | | | | |
| --- | --- | --- | --- | --- | --- | --- | --- |
| **Model** | **Unstandardized coefficients** | | **Standardized coefficients** | ***t*** | **Sig.** | **95% confidence interval for *B*** | |
|  | ***B*** | **SE** | **Beta** |  |  | **LL** | **UL** |
| **Constant** | 2.752 | 0.697 |  | 3.947 | ˂ 0.001 | 1.381 | 4.122 |
| **SR** | -0.317 | 0.126 | -0.172 | -2.522 | 0.012 | -0.563 | -0.070 |
| **EOE** | 0.164 | 0.099 | 0.105 | 1.662 | 0.097 | -0.030 | 0.357 |
| **SE** | -0.325 | 0.102 | -0.188 | -3.174 | 0.002 | -0.527 | -0.124 |
| **Age (y)** | -0.131 | 0.045 | -0.125 | -2.890 | 0.004 | -0.219 | -0.042 |
| **FR** | 0.249 | 0.115 | 0.147 | 2.164 | 0.031 | 0.023 | 0.475 |

Multiple linear regression (stepwise). Number of blocks = 5; F for block = 4.682, *P* = 0.031; adjusted *R*^2^ = 0.26. *BMI* body mass index, *FR* food responsiveness, *EOE* emotional over-eating, *SR* satiety responsiveness, *SE* slowness in eating, *LL* lower limit, *UL* upper limit. ^a^Dependent variable: BMI *Z* score
